# Early pericardial effusion as complication of umbilical venous catheter insertion in extreme preterm baby: A case report

**DOI:** 10.1002/ccr3.3957

**Published:** 2021-02-18

**Authors:** Roya Arif Huseynova, Latifa A. Bin Mahmoud, Morabet AlHemiad, Muath Almuhaini, Oqtay Huseynov

**Affiliations:** ^1^ King Saud Medical City King Saud Medical City Riyadh Saudi Arabia; ^2^ Azerbaijan Medical University Nariman Narimanov Baku Azerbaijan

**Keywords:** central line, neonates, pericardial effusion, pericardiocentesis, preterm, umbilical vein catheter

## Abstract

Reminder essential clinical practice: Pericardial effusion is a rare fatal condition, however potentially reversible when grasped in time. It should always be thought out in neonate with a central line who develops unexplained cardiorespiratory failure.

## Graphical abstract

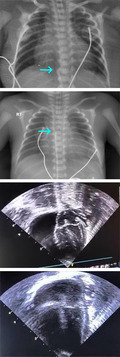

## 1 INTRODUCTION

Umbilical line insertion is one of the frequently performed procedures in neonatal intensive care. However, it is known to be associated with complications ranging from infection to pericardial effusion and subsequent cardiac tamponade.^1^ Pericardial effusion (PCE) and cardiac tamponade (CT) are rare but life‐threatening complications of central venous catheterization due to the abnormal collection of fluid between visceral and parietal layers of the pericardium which in turn predispose to decrease in cardiac output and heart contractility. The incidence of PCE in infants due the central venous catheterization (CVC) use is estimated to be 5%, with mortality rate ranging from 30% to 90%.^2^, ^3^ The advisable position for the tip of an UVC is at the junction of right atrium and inferior vena cava (IVC).^4^ The presence of the tip of the UVC in the cardiac chambers is associated with the risk of pericardial effusion.

Although pericardial effusion is a fatal condition in neonates, however potentially reversible when it is grasped in time. We report a case of early pericardial effusion as a complication from the umbilical venous catheterization diagnosed by urgent echocardiography and followed by immediate pericardiocentesis.

## 2 CASE REPORT

A 995‐g (10th centile) male baby was born at 27 weeks’ gestation by urgent cesarean section because of abruptio placentae to the 30 years old, gravida 2, para 1 mother.

**FIGURE 1:**
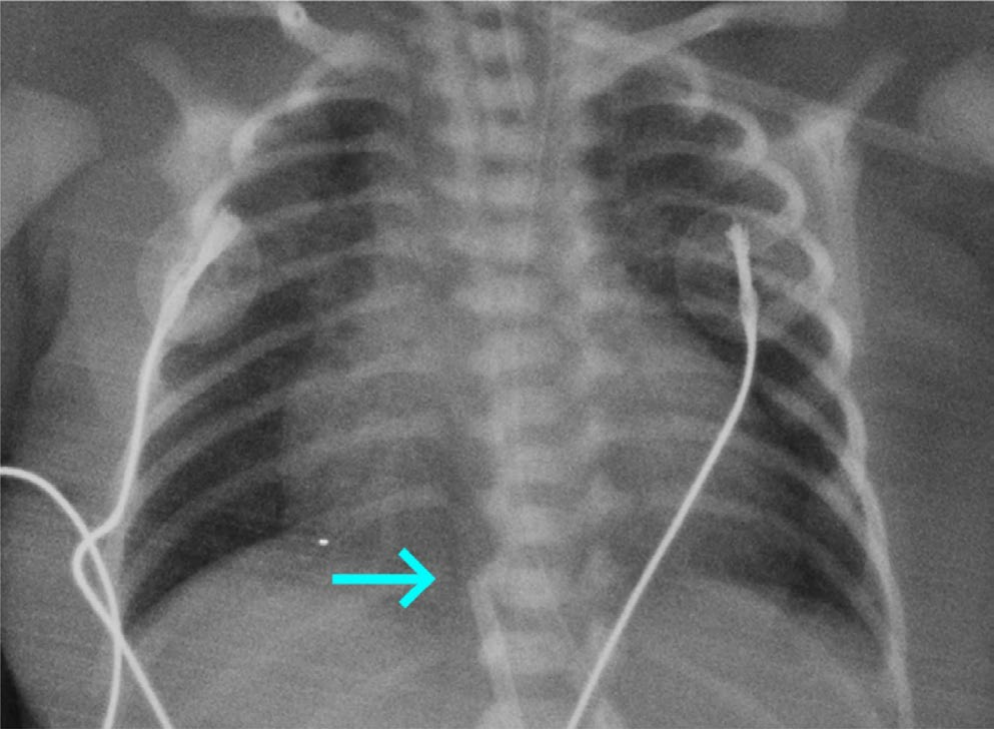
Chest radiography shows the acceptable catheter tip position at the level 10th thoracic vertebrae

Pregnancy was complicated by episodes of vaginal bleeding during the first and third trimesters. Apgar scores were 5 and 7 at 1 and 5 minutes, respectively. The baby was intubated due to respiratory distress, connected to a conventional ventilator, and received Surfactant. Umbilical vein catheter (UVC) size 5 French was inserted at the age of 3 hours by the senior specialist. It was an uneventful procedure, and the position of the UVC tip was confirmed by chest radiograph before the start of parenteral nutrition and medications (Figure 1).

At the age of 5 hours, the patient developed signs of deterioration in the form of desaturation, bluish discoloration of the skin, and dropping blood pressure that required inotropic support.

**FIGURE 2:**
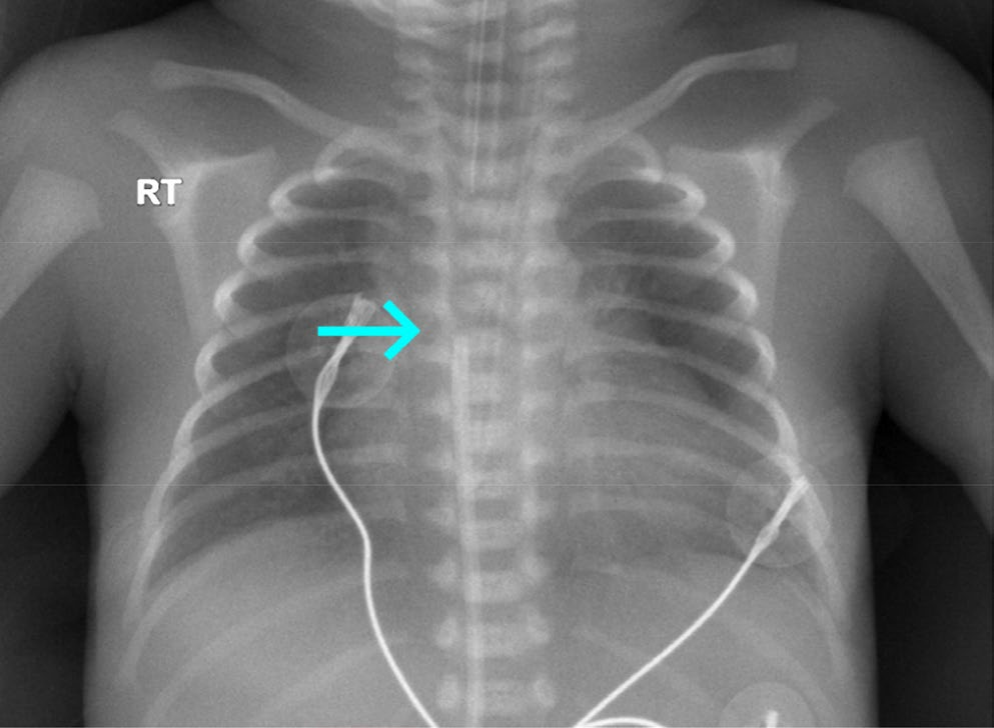
Chest radiography shows the tip of the umbilical venous catheter in the right atrium (displaced)

Chest auscultation revealed equal air entry. The chest radiography (Figure 2) did not reveal pneumothorax or cardiomegaly. The endotracheal tube was identified in the proper position; however, the UVC tip migrated straight upward to the T‐6 vertebral body level, which appeared to be in the right atrium.

All fluids through the UVC were stopped immediately, and the catheter tip was withdrawn into the inferior vena cava shown and confirmed by repeated chest radiography.

The venous blood gas analysis showed a pH: 7.064, partial pressure of carbon dioxide (pC02):79.5 mmHg, and base deficit: ‐ 7.3mEq.

The baby was reintubated because of increasing oxygen requirement and worsening respiratory distress. The ventilatory setting was advanced with minimal improvement. Shortly after, the patient suddenly developed acute bradycardia and active cardiopulmonary resuscitation was performed with administration of intravenous adrenaline. Urgent echocardiography requested and was immediately performed due to the presence of cardiology staff in the unit at that time.

**FIGURE 3:**
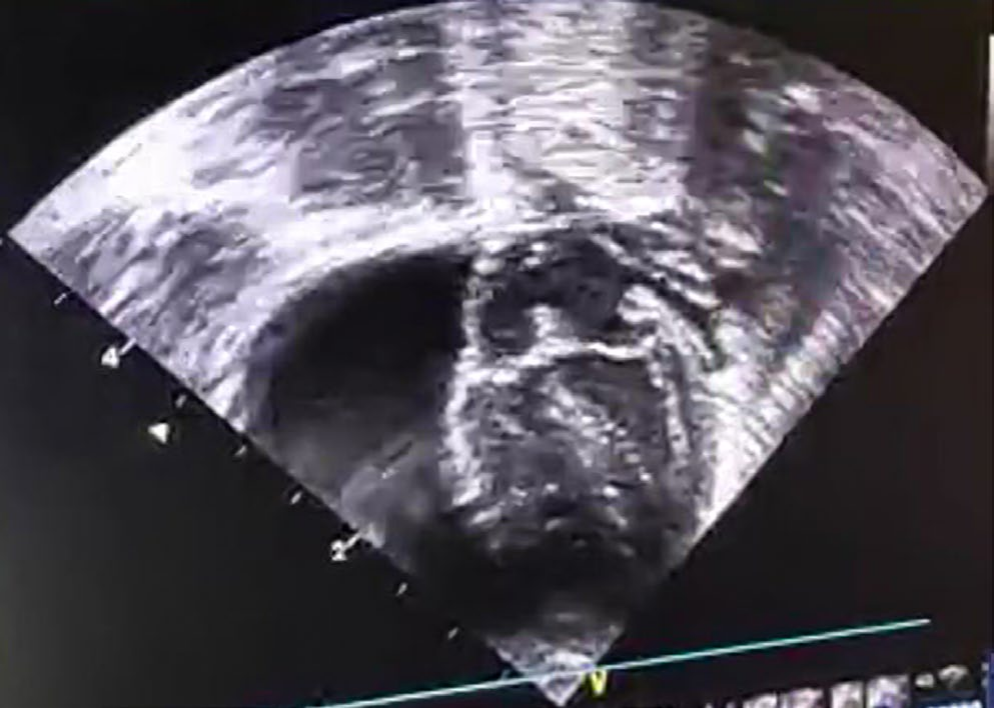
Echocardiography shows the huge pericardial effusion

Bedside 2D echocardiography revealed large amount of pericardial effusion (Figure 3).

Echocardiography‐guided pericardiocentesis was done through a subxiphoid route, and 8‐ml clear fluid was aspirated, resulting in the recovery of circulation. Repeated echocardiography after pericardial tapping revealed minimal pericardial effusion.

**FIGURE 4:**
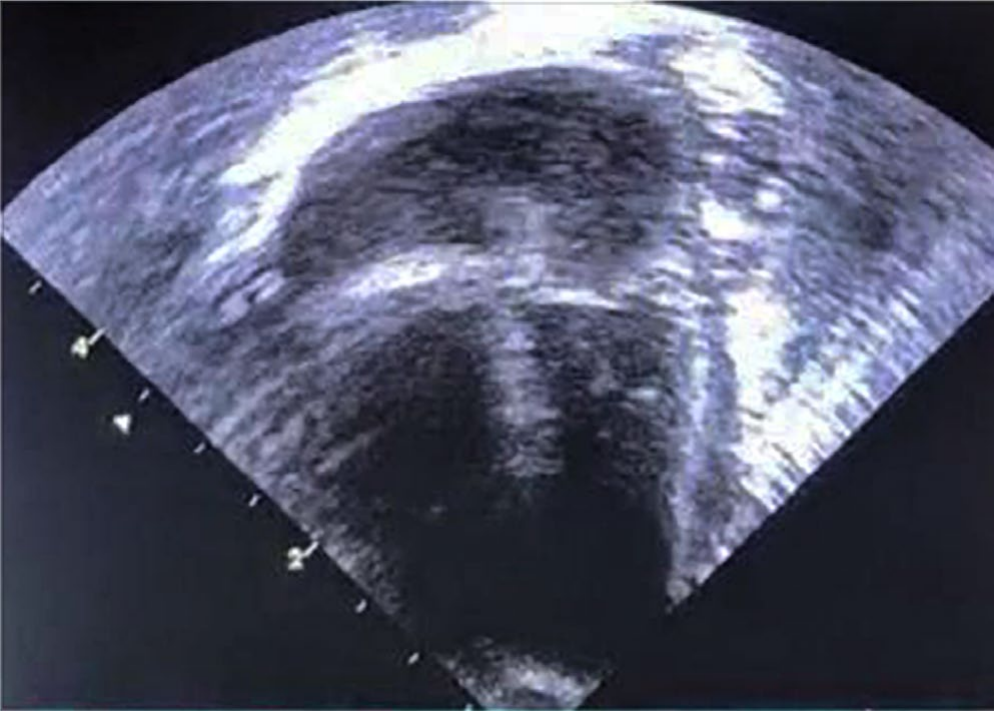
Echocardiography shows resolution of pericardial effusion

Throughout the hospital course, the baby remained hemodynamically stable. Serial echocardiograms showed a gradual reduction of PCE with complete resolution within 3 days (Figure 4).

There was no evidence of reaccumulation thereafter. Currently, the patient is extubated to a high flow nasal cannula, saturating well on 30% FIO2, gaining weight, and on full parenteral feeding.

## 3 DISCUSSION

Central venous catheterization is widely used in the neonatal intensive care unit to provide parenteral nutrition and other solutions for critically ill neonates. Presence of the tip of the UVC in the cardiac chambers considered to be one of the main risk factors for PCE with cardiac tamponade (CT).^5^ Other factors that have been associated with pericardial effusion are extreme preterm infants, misplaced of CVC, and parenteral nutrition infusion.^6^, ^7^ All these risk factors presented in the reported case.

PCE/CT is an uncommon and life‐threatening complication when not promptly diagnosed and expeditiously treated.^8^ It was reported that catheter tip position considered appropriate if it remains outside the cardiac silhouette, approximately 2 cm outside the silhouette in term and 1 cm in preterm neonates.^2^

Direct injury of myocardium wall, hyperosmotic damage that may be caused by infusion of total parenteral nutrition (TPN), and necrosis of myocardium wall due to frequent contact of the myocardium with the tip of the catheter may predispose to PCE.^9^

Most frequently, perforation has a delayed course, and several reports concluded that the average time between placing the catheter and the diagnosis of PCE with CT was 2.5 days.^6^, ^10^

Furthermore, several retrospective studies reported the occurrence of PCE/CT in the presence of a satisfactory position of UVC tip where the occurrence of PCE with cardiac tamponade most probably happened because of the hyperosmolar parenteral nutrition infusion that caused endocardial injury and further penetration of this fluid into the pericardial sac.^11^, ^12^

The acute clinical deterioration that occurred shortly (2 hours) after the insertion of UVC is unlikely related to the osmotic injury of TPN. Migration of the central line and subsequent perforation of the right atrium by UVC tip may be the most plausible explanation of PCE in the reported case where the tip of the catheter was visualized in the right atrium after initial correct positioning.

The migration of the catheter tip is not rare. It was determined by chest radiograph that at 1‐hour post‐UVC insertion, 36% of UVC tip migrated into the cardiac silhouette.^13^

Causes of CVC migration include flushing of the umbilical line by the staff, manipulations, and movements of the extremities or head.^14^

In order to avoid these life‐threatening complications, some reports suggested verifying the position of the catheter tip by chest roentgenogram at least once in 2 days.^15^, ^16^ Also, minimizing the manipulation of neonates with CVC may decrease the risk of displacement of the catheter tip.^17^

Most reported cases of PCE presented with cardiomegaly. Nowlen et al considered increased cardiothoracic ratio as statistically significant findings in PCE (*P* =.001).^10^, ^18^ However, the index case presented an unchanged cardiac silhouette in spite of large amount of PCE.

Many cases of neonates with sudden and unexplained death were diagnosed to have PCE/CT only during the cadaverous examination.^19^, ^20^

Timely diagnosis of PCE and appropriate intervention in the index case was done due to the availability of the cardiac team in the unit at the time of the sudden collapse of the patient that was lifesaving and prevented the possibility of sudden death.

## 4 CONCLUSION

High suspicion of pericardial effusion and cardiac tamponade must be considered in any infant with central line who developed sudden cardiorespiratory collapse. Echocardiography‐guided pericardiocentesis is a key procedure to prevent subsequent mortalities, which has been established by the recovery of the index case. As a result, it is imperative to acknowledge the pressing need for PCE targeted echocardiography training among neonatologists in order to diagnose and subsequently guide treatment in such life‐threatening condition.

## CONFLICT OF INTEREST

None to declare.

## AUTHOR CONTRIBUTIONS

RAH: collected the data, wrote the paper, and critically revised the final manuscript; LBM: prepared and critically revised the article; MFA: collected the data and prepared the primary draft; MSA: collected the data and prepared the primary draft; OIH: collected the data, prepared the primary draft, and critically revised the final manuscript; all authors approved the manuscript as submitted.

## ETHICAL STATEMENT

Informed consent was obtained from parents for reporting this case.

## Data Availability

The data that support the findings of this study are available on request from the corresponding author upon reasonable request.
